# Assisted Identification over Modulo-Additive Noise Channels

**DOI:** 10.3390/e25091314

**Published:** 2023-09-08

**Authors:** Amos Lapidoth, Baohua Ni

**Affiliations:** Signal and Information Processing Laboratory, ETH Zurich, 8092 Zurich, Switzerland; baohni@isi.ee.ethz.ch

**Keywords:** erasures-only capacity, helper, identification capacity, modulo-additive noise, rate-limited, zero-undetected-error capacity

## Abstract

The gain in the identification capacity afforded by a rate-limited description of the noise sequence corrupting a modulo-additive noise channel is studied. Both the classical Ahlswede–Dueck version and the Ahlswede–Cai–Ning–Zhang version, which does not allow for missed identifications, are studied. Irrespective of whether the description is provided to the receiver, to the transmitter, or to both, the two capacities coincide and both equal the helper-assisted Shannon capacity.

## 1. Introduction

If a helper can observe the additive noise corrupting a channel and can describe it to the decoder, then the latter can subtract it and thus render the channel noiseless. However, for this to succeed, the description must be nearly lossless and hence possibly of formidable rate. It is thus of interest to study scenarios where the description rate is limited, and to understand how the rate of the help affects performance.

When performance is measured in terms of the Shannon capacity, the problem was solved for a number of channel models [1,2,3], where the former two address assistance to the decoder and the latter to the encoder. When performance was measured in terms of the erasures-only capacity or the list-size capacity, the problem was solved in [4,5]. Error exponents with assistance were studied in [6]. Here we study how rate-limited help affects the identification capacity [7].

We focus on the memoryless modulo-additive channel (MMANC), whose time-*k* output $Y_{k}$ corresponding to the time-*k* input $x_{k}$ is:

(1) $$Y_{k}=x_{k}⊕Z_{k}$$

where $Z_{k}$ is the time-*k* noise sample; the channel input $x_{k}$, the channel output $Y_{k}$, and the noise $Z_{k}$ all take values in the set A—also denoted X, or Y, or Z—comprising the |A| elements {0,…,|A|−1}; and ⊕ and ⊖ denote mod-|A| addition and subtraction, respectively. The noise sequence $\left\{Z_{k}\right\}$ is IID $∼P_{Z}$, where $P_{Z}$ is some PMF on A.

Irrespective of whether the help is provided to the encoder, to the decoder, or to both, the Shannon capacity of this channel coincides with its erasures-only capacity, and both are given by [3] (Section V) and [4] (Theorems 2 and 6):

(2) $$C_{\text{e-o}}\left(R_{h}\right)=C_{Sh}\left(R_{h}\right)=\log\left|A\right|-{\left\{H\left(P_{Z}\right)-R_{h}\right\}}^{+}$$

where ${\left\{\xi \right\}}^{+}$ denotes max{0,ξ}, and $H(P_{Z})$ is the Shannon entropy of $P_{Z}$.

Here we study two versions of the identification capacity of this channel: Ahlswede and Dueck’s original identification capacity $C_{\mathrm{ID}}$ [7], and the identification capacity subject to no missed-identifications $C_{\mathrm{ID},0}$ [8]. Our main result is that—irrespective of whether the help is provided to the encoder, to the decoder, or to both—the two identification capacities coincide and both equal the right-hand side (RHS) of (2).

## 2. Problem Formulation

The identification-over-a-channel problem is parameterized by the blocklength *n*, which tends to infinity in the definition of the identification capacity. The *n*-length noise sequence $Z^{n}\in A^{n}$ is presented to a helper, which produces its $nR_{h}$-bit description $t(Z^{n})$:

(3) $$t(z^{n})\in T$$

where:

(4) $$T={\left\{0,1\right\}}^{nR_{h}}.$$

We refer to the set N={1,…,N} as the set of identification messages and to its cardinality N as the number of identification messages. The identification rate is defined (for N sufficiently large) as:

(5) $$\frac{1}{n}\log\log N.$$

A generic element of N—namely, a generic identification message—is denoted *i*.

If no help is provided to the encoder, then the latter is specified by a family ${\left\{P_{X^{n}}^{i}\right\}}_{i\in N}$ of PMFs on $A^{n}$ that are indexed by the identification messages, with the understanding that, to convey the identification message (IM) *i*, the encoder transmits a random sequence in $A^{n}$ that it draws according to the PMF $P_{X^{n}}^{i}$. If help $T=t(Z^{n})\in T$ is provided to the encoder, then the encoder’s operation is specified by a family of PMFs ${\left\{P_{X^{n}|t}^{i}\right\}}_{(i,t)\in N\times T}$ that is now indexed by pairs of identification messages and noise descriptions, with the understanding that, to convey IM *i* given the description $T=t(Z^{n})$, the encoder produces a random *n*-length sequence of channel inputs that is distributed according to $P_{X^{n}|T}^{i}$. In either case, the channel output sequence $Y^{n}$ is:

(6) $$Y^{n}=X^{n}⊕Z^{n}$$

componentwise.

If help is provided to the encoder, and if IM *i* is to be conveyed, then the joint distribution of $\left(X^{n},Z^{n},Y^{n},T\right)$ has the form:

(7) $$P_{Z^{n}}\left(z^{n}\right)\;P_{T|Z^{n}}\left(t|z^{n}\right)\;P_{X^{n}|T}^{i}\left(x^{n}|t\right)\;P_{Y^{n}|X^{n},Z^{n}}\left(y^{n}|x^{n},z^{n}\right)$$

where

(8) $$P_{Y^{n}|X^{n},Z^{n}}\left(y^{n}|x^{n},z^{n}\right)=𝟙\left\{y^{n}=x^{n}⊕z^{n}\right\}$$

and where

(9) $$P_{T|Z^{n}}\left(t|z^{n}\right)=𝟙\left\{t=t(z^{n})\right\}$$

because we are assuming that the noise description is a deterministic function of the noise sequence. (The results also hold if we allow randomized descriptions: our coding schemes employ deterministic descriptions and the converse allows for randomization.) Here 𝟙{statement} equals 1 if the statement holds and equals 0 otherwise. In the absence of help, the joint distribution has the form:

(10) $$P_{Z^{n}}\left(z^{n}\right)\;P_{T|Z^{n}}\left(t|z^{n}\right)\;P_{X^{n}}^{i}\left(x^{n}\right)\;P_{Y^{n}|X^{n},Z^{n}}\left(y^{n}|x^{n},z^{n}\right).$$

Based on the data available to it—$Y^{n}$ in the absence of help to the decoder and $\left(Y^{n},t\left(Z^{n}\right)\right)$ in its presence—the receiver performs N binary tests indexed by i∈N, where the *i*-th test is whether or not the IM is *i*. It accepts the hypothesis that the IM is *i* if $Y^{n}$ is in its acceptance region, which we denote $D_{i}\left(t\right)\in A^{n}$ in the presence of decoder assistance t∈T and $D_{i}\in A^{n}$ in its absence.

When the help t∈T is provided to the receiver, the probability of missed detection associated with IM i∈N is thus:

(11) $$p_{\mathrm{MD}}^{i}\left(t\right)=1-P_{Y^{n}|T=t}^{i}\left(D_{i}\left(t\right)\right)$$

and the worst-case false alarm associated with it is:

(12) $$p_{\mathrm{FA}}^{i}\left(t\right)=\max_{j\in N\setminus \{i\}}P_{Y^{n}|T=t}^{j}\left(D_{i}\left(t\right)\right).$$

Note that, given t∈T, the acceptance regions ${\left\{D_{i}\left(t\right)\right\}}_{i\in N}$ of the different tests need not be disjoint. We define:

(13) $$p_{\mathrm{MD},\max}=\max_{i\in N}\sum_{t\in T}P_{T}\left(t\right)\;p_{\mathrm{MD}}^{i}\left(t\right)$$

and:

(14) $$p_{\mathrm{FA},\max}=\max_{i\in N}\sum_{t\in T}P_{T}\left(t\right)\;p_{\mathrm{FA}}^{i}\left(t\right).$$

In the absence of help to the receiver, the probability of missed detection associated with IM *i* is:

(15) $$p_{\mathrm{MD}}^{i}=1-\sum_{t\in T}P_{T}\left(t\right)P_{Y^{n}|T=t}^{i}\left(D_{i}\right)=1-P_{Y^{n}}^{i}\left(D_{i}\right)$$

and the worst-case probability of false alarm associated with it is:

(16) $$p_{\mathrm{FA}}^{i}=\sum_{t\in T}P_{T}\left(t\right)\max_{j\in N\setminus \{i\}}P_{Y^{n}|T=t}^{j}\left(D_{i}\right).$$

In this case, we define:

(17) $$p_{\mathrm{MD},\max}=\max_{i\in N}\;p_{\mathrm{MD}}^{i}$$

and:

(18) $$p_{\mathrm{FA},\max}=\max_{i\in N}\;p_{\mathrm{FA}}^{i}.$$

In both cases we say that a scheme is of zero missed detectionsif $p_{\mathrm{MD},\max}$ is zero.

A rate *R* is an achievable identification rate if, for every γ>0 and every ϵ>0, there exists some positive integer $n_{0}$ such that, for all blocklengths *n* exceeding $n_{0}$, there exists a scheme with:

(19) $$N=\lceil 2^{2^{n(R-\gamma )}}\rceil$$

identification messages for which:

(20) $$\max\{p_{\mathrm{MD},\max},\;p_{\mathrm{FA},\max}\}<\epsilon .$$

The supremum of achievable rates is the identification capacity with a helper $C_{\mathrm{ID}}\left(R_{h}\right)$. Replacing requirement (20) with:

(21) $$p_{\mathrm{MD},\max}=0,\;p_{\mathrm{FA},\max}<\epsilon$$

leads to the definition of the zero missed-identification capacity $C_{\mathrm{ID},0}\left(R_{h}\right)$.

**Remark** **1.** *Writing out $p_{\mathrm{FA},\max}$ of *(14)* as:*

(22) $$p_{\mathrm{FA},\max}=\max_{i\in N}\sum_{t\in T}P_{T}\left(t\right)\;\max_{j\in N\setminus \{i\}}P_{Y^{n}|T=t}^{j}\left(D_{i}\left(t\right)\right)$$

*highlights that (prior to maximizing over i) we first maximize over j and then average the result over t. In this sense, the help—even if provided to both encoder and decoder—cannot be viewed as “common randomness” in the sense of [9,10,11] where the averaging over the common randomness is performed before taking the maximum. Our criterion is more demanding of the direct part (code construction) and less so of the converse.*

*Both criteria are interesting. Ours allows for the notion of “outage”, namely, descriptions that indicate that identification might fail and that therefore call for retransmission. The other criterion highlights the interplay between the noise description and the generation of common randomness (particularly when the help is provided to both transmitter and receiver).*

The following theorem is the main result of this paper.

**Theorem** **1.** *On the modulo additive noise channel—irrespective of whether the help is provided to the transmitter, to the receiver, or to both—the identification capacity with a helper $C_{\mathrm{ID}}\left(R_{h}\right)$ and the zero missed-identification capacity with a helper $C_{\mathrm{ID},0}\left(R_{h}\right)$ are equal and coincide with the Shannon capacity:*

(23) $$C_{\mathrm{ID}}\left(R_{h}\right)=C_{\mathrm{ID},0}\left(R_{h}\right)=C_{\mathrm{Sh}}\left(R_{h}\right)$$

*where the latter is given in *(2)*.*

We prove this result by establishing in Section 3 that $C_{\mathrm{ID},0}\left(R_{h}\right)\geq C_{\mathrm{Sh}}\left(R_{h}\right)$ using a slight strengthening of recent results in [4] in combination with the code construction proposed in [8]. The converse is proved in Section 4, where we use a variation on a theme by Watanabe [12] to analyze the case where the assistance is provided to both transmitter and receiver.

## 3. Direct Part: Zero Missed Detection

In this section we prove that:

(24) $$C_{\mathrm{ID},0}\left(R_{h}\right)\geq C_{\mathrm{Sh}}\left(R_{h}\right)$$

by proposing identification schemes of no missed detections and of rates approaching $C_{\mathrm{Sh}}\left(R_{h}\right)$. To this end, we extend to the helper setting the connection—due to Ahlswede, Cai, and Zhang [8]—between the zero-missed-detection identification capacity $C_{\mathrm{ID},0}$ and the erasures-only capacity $C_{\text{e-o}}$. We then call on recent results [4] to infer that, on the modulo-additive noise channel with a helper, the Erasures-Only capacity is equal to the Shannon capacity. We treat encoder-only assistance and decoder-only assistance separately. Either case also proves achievability when the assistance is provided to both encoder and decoder.

Recall that an erasures-only decoder produces a list L comprising the messages under which the observation is of positive likelihood and then act as follows: If the list contains only one message, it produces that message; otherwise, it declares an erasure. Since the list always contains the transmitted message, this decoder never errs. The erasures-only capacity is defined like the Shannon capacity, but with the additional requirement that the decoder be the erasures-only decoder. This notion extends in a natural way to settings with a helper [4].

### 3.1. Encoder Assistance

A rate-*R*, blocklength-*n*, encoder-assisted, erasures-only transmission code comprises a message set M={1,…,M} with $M=2^{nR}$ messages and a collection of M mappings ${\left\{f_{m}\right\}}_{m\in M}$ from T to $X^{n}$, indexed by M, with the understanding that to transmit Message *m* after being presented with the help $t(Z^{n})\in T$, the encoder produces the *n*-tuple of channel inputs $f_{m}\left(t(Z^{n})\right)\in X^{n}$. Since the decoder observes only the channel outputs (and not the help), it forms the list:

(25) $$L\left(y^{n}\right)=\left\{m\in M:\exists t\in T\;s.t.\;P_{Y^{n}|X^{n},T}\left(y^{n}\left|f_{m}\left(t\right),t\right)\right)>0\right\}.$$

The collection of output sequences that cause the erasures-only decoder to produce an erasure is:

(26) $$Y_{\mathrm{er}}=\left\{y^{n}\in A^{n}:\left|L\left(y^{n}\right)\right|>1\right\}.$$

The probability of erasure associated with the transmission of Message *m* with encoder help *t* is $P_{Y^{n}|X^{n},T}\left(Y_{\mathrm{er}}|f_{m}\left(t\right),t\right)$. On the modulo additive noise channel with rate-$R_{h}$ encoder assistance, the erasures-only capacity and the Shannon capacity coincide and [4]:

(27) $$C_{\text{e-o}}\left(R_{h}\right)=C_{\mathrm{Sh}}\left(R_{h}\right)=\log\left|A\right|-{\left\{H\left(P_{Z}\right)-R_{h}\right\}}^{+}.$$

We shall need the following slightly-stronger version of the achievability part of this result, where we swap the maximization over the messages with the expectation over the help:

**Proposition** **1.** *Consider the modulo additive noise channel with rate-$R_{h}$ encoder assistance. For any transmission rate R smaller than $C_{\text{e-o}}\left(R_{h}\right)$ of *(27)*), there exists a sequence of rate-R transmission codes for which:*

(28) $$\lim_{n\to \infty }\sum_{t\in T}P_{T}\left(t\right)\max_{m\in M}P_{Y^{n}|X^{n},T}\left(Y_{\mathrm{er}}|f_{m}\left(t\right),t\right)=0.$$

*A similar result holds for decoder assistance.*

**Proof.**  The proof is presented in Appendix A. It is based on the construction in [4], but with a slightly finer analysis. □

The coding scheme we propose is essentially that of [8]. We just need to account for the help. For each blocklength *n*, we start out with a transmission code of roughly $2^{nC_{\text{e-o}}\left(R_{h}\right)}$ codewords for which (28) holds, and use Lemma 1 ahead to construct approximately $2^{2^{nC_{\text{e-o}}\left(R_{h}\right)}}$ lightly-intersecting subsets of its message set. We then associate an IM with each of the subsets, with the understanding that to transmit an IM we pick uniformly at random one of the messages in the subset associated with it and transmit this message with the helper’s assistance.

**Lemma** **1** ([7] Proposition 14)**.** *Let Z be a finite set, and let $\lambda \in (0,\frac{1}{2})$ be given. If ϵ>0 is sufficiently small so that:*

(29) $$\lambda \log\left(\frac{1}{\epsilon }-1\right)>2\;\mathrm{and}\;\frac{1}{\epsilon }>6$$

*then there exist subsets $A_{1},\ldots ,A_{N}$ of Z such that for all distinct i,j∈{1,…,N} the following hold:*

(30)(a)|Ai|=ϵ|Z|,(31)(b)|Ai∩Aj|<λϵ|Z|,(32)(c)N≥|Z|−1·2⌊ϵ|Z|⌋−1.

With the aid of this lemma, we can now prove the achievability of $C_{\text{e-o}}\left(R_{h}\right)$.

**Proof.**  Given an erasures-only encoder-assisted transmission code ${\left\{\left(f_{m}\right)\right\}}_{m\in M}$ where $f_{m}:T\to X^{n}$, we apply Lemma 1 to the transmission message set M with:

(33) $$\epsilon =\frac{1}{n^{2}+2}\;\mathrm{and}\;\lambda =\frac{1}{\log n}$$

to infer, for large enough *n*, the existence of subsets $F_{1},\ldots ,F_{N}\subseteq M$ such that for all distinct i,j∈{1,…,N} with j≠i:

(34)|Fi|=Mn2+2(35)|Fi∩Fj|<1lognMn2+2(36)N≥M−1·2Mn2+2−1.

Note that (36) implies that:

(37) $$\underset{n\to \infty }{\underline{\lim}}\left(\frac{1}{n}\log\log N-\frac{1}{n}\log M\right)\geq 0.$$

To send IM *i* after obtaining the assistance $t(z^{n})$, the encoder picks a random element *M* from $F_{i}$ equiprobably and transmits $X^{n}=f_{M}\left(t(Z^{n})\right)$, so:

(38) $$P_{X^{n}|T}^{i}\left(x^{n}|t\right)=\frac{1}{|F_{i}|}\sum_{m\in F_{i}}𝟙\left\{x^{n}=f_{m}\left(t\right)\right\}.$$

To guarantee no missed detections, we set the acceptance region of *i*-th IM to be:

(39) $$D_{i}=\left\{y^{n}\in Y^{n}:\exists \left(m,t\right)\in F_{i}\times T\;s.t.\;P_{Y^{n}|X^{n},T}\left(y^{n}|f_{m}\left(t\right),t\right)>0\right\}.$$

It now remains to analyze the scheme’s maximal false-alarm probability.

(40)pFA,max=maxi∈N∑t∈TPT(t)maxj∈N∖{i}PYn|T=tj(Di)(41)=maxi∈N∑t∈TPT(t)maxj∈N∖{i}1|Fj|∑m∈FjPYn|Xn,TDi|fm(t),t(42)=maxi∈N∑t∈TPT(t)maxj∈N∖{i}1|Fj|[∑m∈Fj∖FiPYn|Xn,TDi|fm(t),t+∑m∈Fj∩FiPYn|Xn,TDi|fm(t),t](43)≤maxi∈N∑t∈TPT(t)maxj∈N∖{i}1|Fj|∑m∈Fj∖FiPYn|Xn,TDi|fm(t),t+Fj∩Fi(44)≤maxi∈N∑t∈TPT(t)maxj∈N∖{i}1|Fj|∑m∈Fj∖FiPYn|Xn,TYer|fm(t),t+Fj∩Fi(45)<maxi∈N∑t∈TPT(t)maxj∈N∖{i}1|Fj|∑m∈Fj∖FiPYn|Xn,TYer|fm(t),t+⌊Mn2+2⌋|Fj|log n(46)≤maxi∈N∑t∈TPT(t)maxj∈N∖{i}Fj∖Fi|Fj|maxm∈MPYn|Xn,TYer|fm(t),t+1log n(47)≤maxi∈N∑t∈TPT(t)maxj∈N∖{i}maxm∈MPYn|Xn,TYer|fm(t),t+1log n(48)=∑t∈TPT(t)maxm∈MPYn|Xn,TYer|fm(t),t+1log n

where in (41) we expressed $P_{Y^{n}|T=t}^{j}\left(D_{i}\right)$ as $P_{Y^{n}|X^{n},T}\left(D_{i}|f_{m}\left(t\right),t\right)$ using (7); in (42) we expressed $F_{j}$ as the disjoint union of $F_{j}\cap F_{i}$ and $F_{j}\setminus F_{i}$; in (43) we used the trivial bound:

(49) $$P_{Y^{n}|X^{n},T}\left(D_{i}|f_{m}\left(t\right),t\right)\leq 1;$$

in (44) we used the fact that whenever m≠i:

(50) $$P_{Y^{n}|X^{n},T}\left(D_{i}|f_{m}\left(t\right),t\right)\leq P_{Y^{n}|X^{n},T}\left(Y_{\mathrm{er}}|f_{m}\left(t\right),t\right)$$

which holds because, by the definition of the set $Y_{\mathrm{er}}$, any output sequence $y^{n}$ that contributes to the LHS of (50), i.e., that is in $D_{i}$ with $P_{Y^{n}|X^{n},T}\left(y^{n}|f_{m}\left(t\right),t\right)>0$, must also be in $Y_{\mathrm{er}}$; in (45) we used (35); in (46) we replaced each term in the sum with the global maximum (over m∈M) and used (34); in (47) we used the trivial bound $|F_{j}\setminus F_{i}\left|\leq \right|F_{j}|$; and in (48) we could simplify the expression because the dependence on *i* and *j* is no longer.

The above construction demonstrates that every transmission scheme that drives $\sum_{t\in T}P_{T}\left(t\right)\max_{m\in M}P_{Y^{n}|X^{n},T}\left(Y_{\mathrm{er}}|f_{m}\left(t\right),t\right)$ to zero induces a zero missed-identification scheme that drives the false-alarm probability to zero. Since the former exists for all rates up to $C_{\text{e-o}}\left(R_{h}\right)$, we conclude, by (37), that $C_{\mathrm{ID},0}\left(R_{h}\right)\geq C_{\text{e-o}}\left(R_{h}\right)$. This, in turn, implies that $C_{\mathrm{ID},0}\left(R_{h}\right)\geq C_{\mathrm{Sh}}\left(R_{h}\right)$ and hence concludes the achievability proof for encoder-assistance because, on the modulo additive noise channel, $C_{\text{e-o}}\left(R_{h}\right)=C_{\mathrm{Sh}}\left(R_{h}\right)$. □

### 3.2. Decoder Assistance

When, rather than to the encoder, the assistance is to the decoder, the transmission codewords are *n*-tuples in $A^{n}$, and we denote the transmission codebook $C={\left\{x^{n}\left(m\right)\right\}}_{m\in M}$. For the induced identification scheme we use the same message subsets as before, with IM *i* being transmitted by choosing uniformly at random a message *M* from the subset $F_{i}$ and transmitting the codeword $x^{n}\left(M\right)$. To avoid any missed detections, we set the acceptance region corresponding to IM *i* and decoder assistance *t* to be:

(51) $$D_{i}\left(t\right)=\{y^{n}\in A^{n}:\exists m\in F_{i}\;s.t.\;P_{Y^{n},T|X^{n}}\left(y^{n},t|x^{n}\left(m\right)\right)>0\}$$

The analysis of the false-alarm probability is nearly identical to that with encoder assistance and is omitted.

## 4. Converse Part: Help Provided to Both Transmitter and Receiver

In this section we establish the converse for all the cases of interest by proving that the inequality:

(52) $$C_{\mathrm{ID}}\left(R_{h}\right)\leq \log\left|A\right|-{\left\{H\left(P_{Z}\right)-R_{h}\right\}}^{+}$$

holds even when the help is provided to both encoder and decoder. The RHS of (52) is the helper Shannon capacity, irrespective of whether the help is provided to the encoder, to the decoder, or to both [3] (Section V).

There are two main steps to the proof. The first addresses the conditional probabilities of the two types of testing errors conditional on a given description T=t. It relates the two to the conditional entropy of the noise given the description, namely, $H(Z^{n}|T=t)$. Very roughly, this corresponds to proving the converse part of the ID-capacity theorem for the channel whose noise is distributed according to the conditional distribution of $Z^{n}$ given T=t. The difficulty in this step is that, given T=t, the noise is not memoryless, and the channel may not even be stable. Classical type-based techniques for proving the converse part of the ID-capacity theorem—such as those employed in [7] (Theorem 12), [13] (Section III), or [14] (Section III)—are therefore not applicable. Instead, we extend to the helper setting Watanabe’s technique [12], which is inspired by the partial channel resolvability method introduced by Steinberg [15].

The second step in the proof addresses the unconditional error probabilities. This step is needed because, in the definition of achievability (see (13) and (14)), the error probabilities are averaged over the noise description *t*. We will show that, when the identification rate exceeds the Shannon capacity, there exists an IM $i^{*}$ for which the sum of the two types of errors is large whenever the description *t* is in a subset $T^{*}$ of T whose probability is bounded away from zero. This will imply that, for this IM $i^{*}$, the sum of the averaged probabilities of error is bounded away from zero, thus contradicting the achievability.

### 4.1. Additional Notation

Given a PMF $P_{X}$ and a conditional PMF $P_{Y|X}$, we write $P_{X}\circ P_{Y|X}$ for the joint PMF that assigns the pair (x,y) the probability $P_{X}\left(x\right)\;P_{Y|X}\left(y|x\right)$. We use $I_{P\circ P_{Y|X}}\left(X;Y\right)$ to denote the mutual information between *X* and *Y* under the joint distribution $P\circ P_{Y|X}$. The product PMF of marginals $P_{X}$ and $P_{Y}$ is denoted $P_{X}\times P_{Y}$; it assigns (x,y) the probability $P_{X}\left(x\right)\;P_{Y}\left(y\right)$.

For the hypothesis testing problem of guessing whether some observation *X* was drawn $∼P_{X}$ (the “null hypothesis”) or $∼Q_{X}$ (the “alternative hypothesis”), we use K(·|X) to denote a generic randomized test that, after observing X=x, guesses the null hypothesis ($X∼P_{X}$) with probability K(0|X=x) and the alternative ($X∼Q_{X}$) with probability K(1|X=x). (Here K(0|X=x)+K(1|X=x)=1 for every x∈X.) The type I error probability associated with K(·|X) is:

(53) $$\lambda_{1}\left[K\right]=\sum_{x\in X}P_{X}\left(x\right)\;K\left(1|x\right)$$

and the type II:

(54) $$\lambda_{2}\left[K\right]=\sum_{x\in X}Q_{X}\left(x\right)\;K\left(0|x\right).$$

For a given 0<ϵ<1 we define:

(55) $$\beta_{\epsilon }\left(P_{X},Q_{X}\right)=\underset{K:\;\lambda_{1}\left[K\right]\leq \epsilon }{\inf}\lambda_{2}\left[K\right]$$

to be the least type-II error probability that can be achieved under the constraint that the type-I error probability does not exceed ϵ.

### 4.2. Conditional Missed-Detection and False-Alarm Probabilities

The following lemma follows directly from Watanabe’s work [12].

**Lemma** **2** ([12] Theorem 1 and Corollary 2)**.** *Let $P_{Y^{n}|X^{n},T=t}$ be the n-letter conditional distribution of the channel output sequence given that the noise description is T=t and the input is $X^{n}$. For any $\lambda_{1}$, $\lambda_{2}$ > 0 with $\lambda_{1}+\lambda_{2}<1$, any $0<\eta <1-\lambda_{1}-\lambda_{2}$, and any fixed t∈T, the condition:*

(56) $$p_{\mathrm{MD}}^{i}\left(t\right)+p_{\mathrm{FA}}^{i}\left(t\right)<\lambda_{1}+\lambda_{2},\;\forall i\in N$$

*implies:*

(57) $$\begin{array}{cc} \log\log N\leq \underset{P\in P(X^{n})}{\sup}\underset{Q\in P(Y^{n})}{\inf} & -\log\beta_{\lambda_{1}+\lambda_{2}+\eta }\left(P\circ P_{Y^{n}|X^{n},T=t},P\times Q\right)\\ & +{\log\log|A|}^{n}+2\log\left(\frac{1}{\eta }\right)+2 \end{array}$$

*and hence:*

(58) $$\frac{1}{n}\log\log N\leq \frac{1}{n}\underset{P\in P(X^{n})}{\sup}\underset{Q\in P(Y^{n})}{\inf}-\log\beta_{\lambda_{1}+\lambda_{2}+\eta }\left(P\circ P_{Y^{n}|X^{n},T=t},P\times Q\right)+\psi_{n}\left(\eta \right),$$

*where:*

(59) $$\psi_{n}\left(\eta \right)=\frac{\log n}{n}+\frac{\log\log|A|}{n}-\frac{2}{n}\log\eta +\frac{2}{n}$$

*which—for any fixed η>0—tends to 0 as n tends to ∞.*

Substituting $P_{Y^{n}|X^{n},T=t}$ for $P_{Y|X}$ in the following theorem will allow us to link the RHS of (57) with the conditional mutual information between $X^{n}$ and $Y^{n}$ given t∈T. The theorem’s proof was inspired by the proof of [16] (Theorem 8). See also [17] (Lemma 1).

**Theorem** **2.** *Given any 0<ϵ<1 and any conditional PMF $P_{Y|X}$,*

(60) $$\underset{P\in P(X)}{\sup}\underset{Q\in P(Y)}{\inf}-\log\beta_{\epsilon }\left(P\circ P_{Y|X},P\times Q\right)\leq \frac{\sup_{P\in P(X)}I_{P\circ P_{Y|X}}\left(X;Y\right)+h\left(\epsilon \right)}{1-\epsilon }$$

*where h(ϵ)≜−ϵlog(ϵ)−(1−ϵ)log(1−ϵ) is the binary entropy function.*

**Proof.**  Applying the data-processing inequality for relative entropy to the binary hypothesis testing setting (see, e.g., [18] (Thm. 30.12.5)) we conclude that for any randomized test K(·|X),

(61) $$D_{\mathrm{bin}}\left(1-\lambda_{1}\left[K\right]\;∥\;\lambda_{2}\left[K\right]\right)\leq D\left(P\circ P_{Y|X}\;∥\;P\times Q\right)$$

where:

(62) $$D_{\mathrm{bin}}\left(\alpha \;∥\;\beta \right)≜\alpha \log\frac{\alpha }{\beta }+\left(1-\alpha \right)\log\frac{1-\alpha }{1-\beta }$$

denotes the binary divergence function. Since there exists a randomized test $K^{*}\left(\cdot |X\right)$ for which $\left(\lambda_{1}\left[K^{★}\right],\lambda_{2}\left[K^{★}\right]\right)=\left(\epsilon ,\beta_{\epsilon }\left(P\circ P_{Y|X},P\times Q\right)\right)$ (see, e.g., [18] (Lemma 30.5.4 and Proposition 30.8.1) we can apply (61) to $K^{★}\left(\cdot |X\right)$ to conclude that:

(63) $$D_{\mathrm{bin}}\left(1-\epsilon \;∥\;\beta_{\epsilon }\left(P\circ P_{Y|X},P\times Q\right)\right)\leq D\left(P\circ P_{Y|X}\;∥\;P\times Q\right).$$

(The above existence also holds when $\beta_{\epsilon }\left(P\circ P_{Y|X},P\times Q\right))$ is zero, but for this case we can verify (63) directly by noting that in this case, since ϵ<1, the RHS of (63) is +∞.) The LHS of (63) can be lower bounded by lower-bounding the binary divergence function as:

(64) $$D_{\mathrm{bin}}\left(1-\epsilon \;∥\;\beta_{\epsilon }\left(P\circ P_{Y|X},P\times Q\right)\right)\geq -h\left(\epsilon \right)-\left(1-\epsilon \right)\log\beta_{\epsilon }\left(P\circ P_{Y|X},P\times Q\right).$$

It follows from (63) and (64) that:

(65) $$-\log\beta_{\epsilon }\left(P\circ P_{Y|X},P\times Q\right)\leq \frac{D\left(P\circ P_{Y|X}\;∥\;P\times Q\right)+h\left(\epsilon \right)}{1-\epsilon }$$

so the infimum over *Q* of the LHS is upper bounded by the infimum over *Q* on the RHS. The latter (for fixed P∈P(X)) is achieved when *Q* is the *Y*-marginal of $P\circ P_{Y|X}$, a marginal that we denote $P_{Y}$:

(66) $$\underset{Q}{\inf}D\left(P\circ P_{Y|X}\;∥\;P\times Q\right)=I_{P\circ P_{Y|X}}\left(X;Y\right).$$

This is a special case of a more general result on Rényi divergence [19] (Theorem II.2). Here we give a simple proof for K-L divergence:

DP∘PY|X∥P×Q(67)=∑x∈X,y∈YP∘PY|X(x,y)logP∘PY|X(x,y)P(x)Q(y)(68)=∑x∈X,y∈YP∘PY|X(x,y)logP∘PY|X(x,y)P(x)PY(y)PY(y)Q(y)(69)=∑x∈X,y∈YP∘PY|X(x,y)logP∘PY|X(x,y)P(x)PY(y)+∑yPY(y)logPY(y)Q(y)(70)≥IP∘PY|X(X;Y)+0

with equality if and only if *Q* equals $P_{Y}$.

From (63), (64), and (66) we obtain:

supP∈P(X)infQ∈P(Y)−logβϵ(P∘PY|X,P×Q)(71)≤supP∈P(X)infQ∈P(Y)Dbin1−ϵ∥βϵ(P∘PY|X,P×Q)+h(ϵ)1−ϵ(72)≤supP∈P(X)infQ∈P(Y)DP∘PY|X∥P×Q+h(ϵ)1−ϵ(73)=supP∈P(X)IP∘PY|X(X;Y)+h(ϵ)1−ϵ.

□

Applying Lemma 2 and Theorem 2 to our channel when its law is conditioned on T=t yields the following corollary.

**Corollary** **1.** *On the* MMANC*, for any $\lambda_{1}$, $\lambda_{2}$ > 0 with $\lambda_{1}+\lambda_{2}<1$, any $0<\eta <1-\lambda_{1}-\lambda_{2}$, and any fixed t∈T, the condition:*

(74) $$p_{\mathrm{MD}}^{i}\left(t\right)+p_{\mathrm{FA}}^{i}\left(t\right)<\lambda_{1}+\lambda_{2},\;\forall i\in N$$

*implies:*

(75) $$\frac{1}{n}\log\log N\leq \frac{\log|A|-H(Z^{n}|T=t)/n}{1-\epsilon^{\prime }}+\psi_{n}\left(\eta \right),$$

*where $\epsilon^{\prime }=\lambda_{1}+\lambda_{2}+\eta$.*

**Proof.**  Substituting $X^{n}$ for X, $Y^{n}$ for Y, $P_{Y^{n}|X^{n},T=t}$ for $P_{Y|X}$, and $\epsilon^{\prime }$ for ϵ in Theorem 2, we obtain:

(76) $$\begin{array}{c} \underset{P\in P(X^{n})}{\sup}\underset{Q\in P(Y^{n})}{\inf}-\log\beta_{\epsilon^{\prime }}\left(P\circ P_{Y^{n}|X^{n},T=t},P\times Q\right)\\ \;\;\leq \frac{\sup_{P\in P(X^{n})}I_{P\circ P_{Y^{n}|X^{n},T=t}}\left(X^{n};Y^{n}\right)+h\left(\epsilon^{\prime }\right)}{1-\epsilon^{\prime }}. \end{array}$$

Given $P\in P(X^{n})$ and $P_{Y^{n}|X^{n},T=t}$, the mutual information term in (76) can be upper-bounded as follows:

(77)IP∘PYn|Xn,T=t(Xn;Yn)≤nlog|A|−HP∘PYn|Xn,T=t(Yn|Xn,T=t)(78)=nlog|A|−∑xnP(xn|T=t)H(Yn|Xn=xn,T=t)(79)=nlog|A|−∑xnP(xn|T=t)H(Zn|Xn=xn,T=t)(80)=nlog|A|−H(Zn|T=t).

Applying (76) and (80) to (58) in Lemma 2 establishes Corollary 1. □

### 4.3. Averaging over *T*

Corollary 1 deals with identification for a given fixed T=t, but our definition of achievability in (13) and (14) entails averaging over *t*, which we must thus study. We begin by lower-bounding the conditional entropy of the noise sequence $Z^{n}$ given the assistance *T*:

(81)H(Zn|T)=H(Zn,T)−H(T)(82)≥{H(Zn)−nRh}+(83)=n{H(PZ)−Rh}+.

We next define, for every δ>0, the subset of descriptions:

(84) $$T^{*}\left(\delta \right)=\left\{t\in T:H\left(Z^{n}|T=t\right)\geq n{\left\{H\left(P_{Z}\right)-R_{h}-\delta \right\}}^{+}\right\}.$$

These are poor noise descriptions in the sense that, after they are revealed, the remaining uncertainty about the noise is still large. Key is that their probability is bounded away from zero. In fact, as we next argue:

(85) $$P_{T}\left(T^{*}\left(\delta \right)\right)\geq \left\{\begin{array}{cc} \frac{\delta }{\log|A|-H\left(P_{Z}\right)+R_{h}+\delta }\; & \mathrm{if}\;R_{h}<H\left(P_{Z}\right)-\delta \\ 1 & \mathrm{if}\;R_{h}\geq H\left(P_{Z}\right)-\delta \end{array}\right.$$

where in the second case the probability is 1 because when $R_{h}\geq H\left(P_{Z}\right)-\delta$ the condition appearing in the definition of $T^{*}\left(\delta \right)$ in (84) translates to $H(Z^{n}|T=t)\geq 0$. As to the first case, we begin with (83) to obtain:

(86)n(H(PZ)−Rh)≤H(Zn|T)(87)=∑t∉T*(δ)PT(t)H(Zn|T=t)+∑t∈T*(δ)PT(t)H(Zn|T=t)(88)≤1−PTT*(δ)·nH(PZ)−Rh−δ+PTT*(δ)·nlog|A|

from which the first case of the bound in (85) follows. Here (87) follows from expressing T as the disjoint union of $T^{*}\left(\delta \right)$ and $T\setminus T^{*}\left(\delta \right)$, and (88) follows from the definition of $T^{*}\left(\delta \right)$ and the bound $H(Z^{n}|T=t)\leq n\log|A|$.

Inequality (85) establishes that the probability of a poor description is lower bounded by a positive constant that does not depend on *n*. Using Corollary 1 for such *t*’s will be the key to the converse.

Henceforth, we fix some sequence of identification codes of rate *R* exceeding $C_{\mathrm{Sh}}\left(R_{h}\right)$, i.e., satisfying $R>\log|A|-{\left\{H\left(P_{Z}\right)-R_{h}\right\}}^{+}$, and show that $p_{\mathrm{MD},\max}+p_{\mathrm{FA},\max}$ cannot tend to 0 as *n* tends to *∞*. For such a rate *R*, there exist $R^{\prime }$, δ>0; a pair $\lambda_{1},\lambda_{2}>0$ with $\lambda_{1}+\lambda_{2}<1$; and some $\eta \in (0,1-\lambda_{1}-\lambda_{2})$ such that:

(89) $$R>R^{\prime }>\frac{\log|A|-{\left\{H\left(P_{Z}\right)-R_{h}-\delta \right\}}^{+}}{1-\epsilon^{\prime }}$$

where $\epsilon^{\prime }≜\lambda_{1}+\lambda_{2}+\eta <1$. Fix such $R^{\prime }$, δ, $\lambda_{1}$, $\lambda_{2}$, η, and $\epsilon^{\prime }$.

Since the inequality on $R^{\prime }$ in (89) is strict, and since $\psi_{n}\left(\eta \right)$ tends to zero with *n*, it follows that the inequality continues to hold also when we add $\psi_{n}\left(\eta \right)$ to the RHS provided that *n* is sufficiently large, i.e., that there exists some $n_{0}\left(\eta \right)$ such that:

(90) $$R^{\prime }>\frac{\log|A|-{\left\{H\left(P_{Z}\right)-R_{h}-\delta \right\}}^{+}}{1-\epsilon^{\prime }}+\psi_{n}\left(\eta \right),\;n\geq n_{0}\left(\eta \right).$$

It then follows from (90) and the definition of $T^{*}\left(\delta \right)$ in (84) that, whenever $n\geq n_{0}\left(\eta \right)$, $R^{\prime }$ exceeds the RHS of (75):

(91) $$R^{\prime }>\frac{\log|A|-n^{-1}\;H\left(Z^{n}|T=t\right)}{1-\epsilon^{\prime }},\;\forall t\in T^{*}\left(\delta \right).$$

Corollary 1 thus implies that, for $n>n_{0}\left(\eta \right)$:

(92) $$\left(\frac{1}{n}\log\log N>R^{\prime }\right)\Rightarrow \left(\forall t\in T^{★}\;\exists i\left(t\right)\in N\;s.t.\;p_{\mathrm{MD}}^{i}\left(t\right)+p_{\mathrm{FA}}^{i}\left(t\right)\geq \lambda_{1}+\lambda_{2}\right).$$

However, we need a stronger statement because, in the above, the IM *i* for which $p_{\mathrm{MD}}^{i}\left(t\right)+p_{\mathrm{FA}}^{i}\left(t\right)\geq \lambda_{1}+\lambda_{2}$ depends on *t*, whereas in our definition of achievability we are averaging over *T* for fixed IM. The stronger result we will establish is that the condition on the LHS of (92) implies that, for all sufficiently large *n*, there exists some IM $i^{*}$ (that does not depend on *t*) which performs poorly for *every t* in $T^{*}\left(\delta \right)$, i.e., for which:

(93) $$p_{\mathrm{MD}}^{i^{*}}\left(t\right)+p_{\mathrm{FA}}^{i^{*}}\left(t\right)\geq \lambda_{1}+\lambda_{2},\;\forall t\in T^{*}\left(\delta \right).$$

That is, we will show that for sufficiently large *n*:

(94) $$\left(\frac{1}{n}\log\log N>R^{\prime }\right)\Rightarrow \left(\exists i\in N\;s.t\;\min_{t\in T^{★}\left(\delta \right)}\left\{p_{\mathrm{MD}}^{i}\left(t\right)+p_{\mathrm{FA}}^{i}\left(t\right)\right\}\geq \lambda_{1}+\lambda_{2}\right).$$

To this end, define for each $t\in T^{*}\left(\delta \right)$:

(95) $$N\left(t\right)=\left\{i\in N:p_{\mathrm{MD}}^{i}\left(t\right)+p_{\mathrm{FA}}^{i}\left(t\right)<\lambda_{1}+\lambda_{2}\right\}$$

and consider the identification code that results when we restrict our code to the IMs in N(t) (while keeping the same acceptance regions). Applying Corollary 1 to this restricted code using (91), we obtain that:

(96) $$\frac{1}{n}\log\log\left|N\left(t\right)\right|<R^{\prime },\;\forall t\in T^{*}\left(\delta \right).$$

Consequently,

(97) $$\left|\underset{t\in T^{*}\left(\delta \right)}{⋃}N\left(t\right)\right|\leq \sum_{t\in T^{*}\left(\delta \right)}\left|N(t)\right|\leq 2^{nR_{h}}\;2^{2^{nR^{\prime }}}$$

where the second inequality holds by (96) and the fact that $T^{*}\left(\delta \right)$ is contained in T, and the latter’s cardinality is $2^{nR_{h}}$.

Since $R^{\prime }<R$ (89), there exists some $n_{1}\left(R,R^{\prime },R_{h}\right)$ such that:

(98) $$2^{nR_{h}}2^{2^{nR^{\prime }}}<2^{2^{nR}},\;n\geq n_{1}\left(R,R^{\prime },R_{h}\right).$$

We can use this to upper-bound the RHS of (97) to obtain that, for $n\geq \max\left\{n_{0}\left(\eta \right),n_{1}\left(R,R^{\prime },R_{h}\right)\right\}$:

(99) $$\left|\underset{t\in T^{*}\left(\delta \right)}{⋃}N\left(t\right)\right|<N.$$

The complement (in N) of the union on the LHS of (99) is thus not empty, which proves the existence of some $i^{★}\in N$ for which (93) holds.

With $i^{★}$ in hand, the converse follows from the fact that the probability that *T* is in $T^{*}\left(\delta \right)$ is bounded away from zero (85), because for every $n\geq \max\left\{n_{0}\left(\eta \right),n_{1}\left(R,R^{\prime },R_{h}\right)\right\}$:

(100)pMD,max+pFA,max=maxi∈N∑t∈TPT(t)pMDi(t)+maxi∈N∑t∈TPT(t)pFAi(t)(101)≥∑t∈TPT(t)pMi*D(t)+pFAi*(t)(102)≥∑t∈T*(δ)PT(t)pMi*D(t)+pFAi*(t)(103)≥∑t∈T*(δ)PT(t)·(λ1+λ2)(104)=PT(T*(δ))·(λ1+λ2)

where (100) follows from the definitions in (13) and (14); in (101) we replaced the maximum with the IM $i^{*}$; and (103) follows from (93). Thus, any code of rate $R>\log|A|-{\left\{H\left(P_{Z}\right)-R_{h}\right\}}^{+}$ with large enough *n* must have $p_{\mathrm{MD},\max}+p_{\mathrm{FA},\max}\geq P_{T}\left(T^{*}\right)\cdot \left(\lambda_{1}+\lambda_{2}\right)$, and the latter is bounded away from zero. This concludes the proof of the converse part.

## Data Availability

Not applicable.

## Abbreviations

The following abbreviations are used in this manuscript:

MMANC	Memoryless modulo-additive noise channel
IID	Identical independent distribution
IM	Identification message
LHS	Left hand side
RHS	Right hand side

## Appendix A. Proof of Proposition 1

**Proof.** As in [4], the code construction entails time-sharing between two schemes: a “zero-rate help scheme” corresponding to help of zero rate, and a “high-rate help scheme” corresponding to help of a rate exceeding the noise entropy. In the former, the help comprises one bit, indicating whether or not the noise is typical. We denote this help $T^{\left(z\right)}$ and assume that it takes values in the set H={τ,α}, with $T^{\left(z\right)}=\tau$ indicating that the noise is typical and $T^{\left(z\right)}=\alpha$ that it is atypical (when the help is to the encoder, the helper additionally provides the encoder with the description of one noise sample in order to enable the encoder to convey $T^{\left(z\right)}$ to the decoder error free). When the help is of high rate, we denote it $T^{\left(h\right)}$. It has two parts, that we denote $T_{t/a}^{\left(h\right)}$ and $T_{d}^{\left(h\right)}$, so $T^{\left(h\right)}=\left(T_{t/a}^{\left(h\right)},T_{d}^{\left(h\right)}\right)$. The first part, $T_{t/a}^{\left(h\right)}$, indicates whether or not the noise is typical and hence takes values in H. The second part, $T_{d}^{\left(h\right)}$, describes the noise (perfectly) when the latter is typical, and is null otherwise (as above, when the help is to the encoder, the helper additionally provides the encoder with the description of one noise sample in order to enable the encoder to convey $T_{t/a}^{\left(h\right)}$ to the decoder error free). The help in the time-sharing scheme, which we denote *T*, comprises the help in the zero-rate part and the help in the high-rate part:

(A1) $$T=\left(T^{\left(z\right)}\;,\;T^{\left(h\right)}\right)$$

The duty cycle is chosen so that the rate of *T* be $R_{h}$ (or the entropy of the noise, if the latter is smaller than $R_{h}$). We assume throughout that R<log|A|. The transmission code derived in [4] has two salient properties:

- In the high-rate scheme, conditional on $T_{t/a}^{\left(h\right)}=\tau$ (i.e., on the noise being typical and that it can therefore be perfectly described by $T_{d}^{\left(h\right)}$), no erasures are declared.
- In the zero-rate scheme, conditional on $T^{\left(z\right)}=\tau$ (i.e., on the noise being typical), the maximal (over the messages) probability of erasure is upper bounded by some $\epsilon_{n}$ tending to zero.

(To guarantee the second property, the code is constructed—as in [4]—using random coding and we then expurgate half the codewords to obtain a code whose maximal probability of erasure is smaller than ϵ. The asserted property then follows by bounding, for each message, the conditional probability of erasure given that the noise is typical by the ratio of the unconditional probability of erasure to the probability that the noise is typical.) We next analyze the time-sharing scheme. We focus on the case where $0<R_{h}\leq H\left(P_{Z}\right)$. The remaining cases, where $R_{h}=0$ or $R_{h}>H\left(P_{Z}\right)$ are very similar, except that they require no time sharing. We use the superscript (h) for quantities occurring in the high-rate help phase, and the superscript (z) for those in the zero-rate phase. For example, $m^{\left(h\right)},X^{\left(h\right)},Y^{\left(h\right)},T^{\left(h\right)}$ are the message, input sequence, output sequence, and help in the high-rate help phase; and the set of output sequences causing an erasure in this phase is denoted $Y_{\mathrm{er}}^{\left(h\right)}$. The set of outputs causing an erasure in the time-sharing scheme is:

(A2) $$Y_{\mathrm{er}}=\left\{y^{n}:y^{\left(h\right)}\in Y_{\mathrm{er}}^{\left(h\right)}\;\mathrm{or}\;y^{\left(z\right)}\in Y_{\mathrm{er}}^{\left(z\right)}\right\}.$$

For the time-sharing scheme we now have:

(A3)∑t∈TPT(t)maxm∈MPYn|Xn,TYer|fm(t),t≤∑t∈TPT(t)maxm∈M[PY(h)|Y(h),T(h)Yer(h)|fm(h)(h)(t(h)),t(h)+PY(z)|X(z),T(z)Yer(z)|fm(z)(z)(t(z)),t(z)](A4)≤∑t∈TPT(t)maxm(h)∈M(h)PY(h)|X(h),T(h)Yer(h)|fm(h)(h)(t(h)),t(h)+∑t∈TPT(t)maxm(z)∈M(z)PY(z)|X(z),T(z)Yer(z)|fm(z)(z)(t(z)),t(z)(A5)=∑t(h))PT(h)(t(h))maxm(h)∈M(h)PY(h)|X(h),T(h)Yer(h)|fm(h)(h)(t(h)),t(h)+∑t(z)PT(z)(t(z))maxm(z)∈M(z)PY(z)|X(z),T(z)Yer(z)|fm(z)(z)(t(z)),t(z)(A6)≤PTt/a(h)=α+PT(z)=α·1+PT(z)=τ·ϵn(A7)≤PTt/a(h)=α+PT(z)=α+ϵn

which establishes the proposition, because the RHS tends to zero. Here (A3) follows from (A2) and the union-of-events bound; (A4) holds (in this case with equality) because the maximum of a sum is upper bounded by the sum of the maxima; and (A6) holds by the aforementioned salient properties of the code construction. □
